# Comparison of vertical jump and sprint performances between 3 × 3 and 5 × 5 elite professional male basketball players

**DOI:** 10.3389/fspor.2024.1394739

**Published:** 2024-05-10

**Authors:** Dimitrije Cabarkapa, Quincy R. Johnson, Jelena Aleksic, Damjana V. Cabarkapa, Nicolas M. Philipp, Marko Sekulic, Darko Krsman, Nenad Trunic, Andrew C. Fry

**Affiliations:** ^1^ Jayhawk Athletic Performance Laboratory—Wu Tsai Human Performance Alliance, Department of Health, Sport and Exercise Sciences, University of Kansas, Lawrence, KS, United States; ^2^ Faculty of Sport and Physical Education, University of Belgrade, Belgrade, Serbia; ^3^ Besiktas Basketball Club, Istanbul, Türkiye; ^4^ International Strength and Conditioning Institute, Novi Sad, Serbia; ^5^ Faculty of Physical Education and Sports Management, Singidunum University, Belgrade, Serbia

**Keywords:** force, power, acceleration, deceleration, eccentric, concentric, sport, monitoring

## Abstract

Given its fast-growing popularity and unique on-court competitive demands, 3 × 3 basketball has captured a considerable amount of attention over recent years. However, unlike research focused on studying 5 × 5 basketball players, there is a lack of scientific literature focused on examining countermovement vertical jump (CMJ) and sprint performance characteristics of 3 × 3 athletes. Thus, the purpose of the present study was to compare force-time metrics during both eccentric and concentric phases of the CMJ and acceleration and deceleration capabilities between 3 × 3 and 5 × 5 top-tier professional male basketball athletes. Ten 3 × 3 and eleven 5 × 5 professional basketball players volunteered to participate in the present study. Upon completion of a standardized warm-up, each athlete performed three maximum-effort CMJs, followed by two 10 m sprints. A uni-axial force plate system sampling at 1,000 Hz was used to analyze CMJ force-time metrics and a radar gun sampling at 47 Hz was used to derive sprint acceleration-deceleration measures. Independent t-tests and Hedge's g were used to examine between-group statistically significant differences (*p* < 0.05) and effect size magnitudes. The findings of the present study reveal that 3 × 3 and 5 × 5 professional male basketball players tend to display similar neuromuscular performance characteristics as no significant differences were observed in any force-time metric during both eccentric and concentric phases of the CMJ (*g* = 0.061–0.468). Yet, prominent differences were found in multiple measures of sprint performance, with large effect size magnitudes (*g* = 1.221–1.881). Specifically, 5 × 5 basketball players displayed greater average and maximal deceleration and faster time-to-stop than their 3 × 3 counterparts. Overall, these findings provide reference values that sports practitioners can use when assessing athletes' CMJ and sprint performance capabilities as well as when developing sport-specific training regimens to mimic on-court competitive demands.

## 1 Introduction

The rapid growth of 3 × 3 basketball in recent years has propelled this game to a global scale, leading to its inclusion in the 2020 Tokyo Olympics (1). When compared to the traditional 5 × 5 competitive format, the 3 × 3 basketball game has notable differences in rules and regulations (2). Besides the evident decrease in the number of players (i.e., 3 starters and 1 reserve), the 3 × 3 game is played on one-half of the standard-sized basketball court (i.e., 15 m width × 11 m length) with a single hoop (3, 4). It is characterized by the fast-paced style of play, involving repetitive high-intensity movements carried out within a 12s shot clock (1). Moreover, 3 × 3 basketball features 10 min of live competitive play with no breaks after the basket is scored, resulting in rapid changes in offensive and defensive possessions, creating a thrilling spectacle for a broad audience spectrum (3, 5).

Given its fast-growing popularity and unique on-court competitive demands, it comes as no surprise that 3 × 3 basketball has captured a considerable amount of attention from sports scientists over recent years (6). However, being a relatively new research topic, the data pertaining to the physical performance characteristics of this group of athletes is limited, particularly at the elite level of 3 × 3 competition (5, 7, 8, 9). Despite some assumptions that the physiological demands of 3 × 3 basketball are similar to the traditional 5 × 5 style of play, there is insufficient evidence supporting the generalization of findings from one game format to another (6, 9). This is particularly noteworthy considering the differences in player count, court dimensions, and rest intervals as factors that can have a substantial impact on athletes' physical and physiological responses and adaptations (10).

Based on a recently published review by Sansone et al. (9), it is obvious that 3 × 3 basketball is distinctly different from classic 5 × 5 basketball. A few research reports suggest that physical and physiological demands are notably higher in 3 × 3 basketball when compared to the 5 × 5 traditional style of play (1, 11, 12). For example, Willberg et al. (12) found that 3 × 3 basketball players tend to perform more medium and high-intensity accelerations and decelerations, jumps, and change-of-direction movements per minute of play than 5 × 5 athletes. In addition, alongside the notably lower work-to-rest ratio, 3 × 3 basketball players tend to spend more playing time in heart rate Zone 5 (i.e., >90% of maximal heart rate) (1, 3, 11, 13). In this context, both male and female 3 × 3 basketball players experience substantially high average heart rates, 165 ± 18 and 164 ± 12 bpm, respectively (6). On the other hand, Figueira et al. (5) found negligible differences in physiological responses between 3 × 3 and 5 × 5 competitive formats in amateur basketball players. The only notable distinction between the two styles of play was found in technical-tactical variables, where players during the 3 × 3 competitive format attained a greater number of ball touches, dribble drives, and long-distance shots (5). Thus, these findings imply the need for further research to differentiate factors that contribute to success in 3 × 3 basketball.

Physical performance characteristics of 5 × 5 basketball players have been extensively studied in the scientific literature (14–18), particularly vertical jump and sprint capabilities (1, 19). The countermovement vertical jump (CMJ) performed on a force plate has commonly been used to obtain a deeper insight into athletes' neuromuscular performance characteristics (20). This is especially beneficial for monitoring changes in various force-time metrics within both the eccentric and concentric phase of the CMJ as well as differentiating basketball players based on their movement strategy and playing position (17, 18, 21). Also, short-distance sprints have been one of the standard testing modalities used in basketball-specific settings to assess speed-related metrics, including the players' ability to accelerate, decelerate, and generate maximal sprint velocity (17, 22, 23). However, despite a considerable number of research reports focused on examining the CMJ and sprint performance parameters of 5 × 5 basketball players (17, 18, 21, 23), there is still a lack of research focused on examining the same performance parameters of 3 × 3 players.

Therefore, the purpose of the present study was twofold: (a) to provide coaches, strength and conditioning practitioners, and sports scientists with a deeper insight into neuromuscular and sprint performance characteristics of 3 × 3 professional male basketball players and (b) to compare force-time metrics during both eccentric and concentric phases of the CMJ and acceleration and deceleration capabilities between 3 × 3 and 5 × 5 professional male basketball players.

## 2 Materials and methods

### 2.1 Participants

Ten 3 × 3 and eleven 5 × 5 basketball players volunteered to participate in the present study. The 3 × 3 athletes examined in this investigation were top-ranked players and members of an official national team that reached the final stages of international tournaments and the 5 × 5 athletes competed at the National Basketball Association (NBA) or top-tier professional basketball leagues in Europe (e.g., EuroLeague, ABA League). All athletes were in between professional contracts or were an active part of the team at the time of the data collection. Also, all athletes were free of musculoskeletal injuries that could limit or impair CMJ and sprint performance. The testing procedures performed in this investigation were previously approved by the University's Institutional Review Board and all participants signed an informed consent document.

### 2.2 Procedures

The testing procedures were conducted in the off-season competitive period. Prior to the start of the data collection, all athletes were thoroughly familiarized with the testing protocols. Each athlete was allowed to perform three CMJ and two sprint practice trials. The athletes did not participate in any type of high-intensity exercise 48 h before the testing procedures.

Upon arrival at the testing facility (12:00–15:00 h), athletes completed a standardized warm-up protocol composed of dynamic stretching exercises (e.g., A-skips, butt-kicks, high knees, side-to-side lunges, high-knee-pulls) (21). Then, each athlete stepped on a dual uni-axial force plate system (ForceDecks Max, VALD Performance, Brisbane, Australia) sampling at 1,000 Hz and performed three maximum-effort CMJs with no arm swing (i.e., hands on the hips during the entire movement). The athletes were instructed to focus on pushing the ground as forcefully as possible (24). To minimize the possible influence of fatigue, each jump was separated by a 10–15 s rest interval. If the athlete accidentally used an arm swing or landed with one or two feet off the force plates, the jump trial was repeated. The system was re-calibrated between each athlete and the mean value across three jump trials was used for performance analysis purposes.

Following the completion of the CMJ testing procedures, each athlete performed two non-consecutive 10 m sprints (i.e., acceleration-deceleration assessment) (18, 25–27). The start position (0 m) was marked with a set of cones and the athletes were instructed to stand still in a staggered stance position. A radar device (Stalker ATS II, Applied Concepts, Inc., Dallas, TX, USA) mounted on a tripod was positioned 5 m behind the start line (0 m), according to the manufacturer's recommendations. The sampling frequency of the radar was 47 Hz. The target direction on the radar was set to “both” to enable the device to record movement going away and toward the radar and the height of the radar was adjusted to be in line with the athlete's estimated center of mass (lower back/hip region). Then, following a “3-2-1-go” command, athletes were instructed to rapidly accelerate, and sprint as fast as possible through the second set of cones (10 m). After crossing the 10 m mark, athletes were instructed to decelerate as rapidly as possible, come to a full stop, stand still for 2–3 s, and then backpedal to the 10 m mark. Each athlete completed two acceleration-deceleration assessments, separated by a 4–5 min rest interval. The average value across two sprint trials was used for performance analysis purposes. If the athlete started to decelerate before crossing the 10 m mark, the acceleration-deceleration assessment was repeated. Also, the two research assistants were present throughout all testing procedures to provide strong verbal encouragement and ensure that athletes were giving maximal effort on each test. Following the completion of the testing procedures the athletes' ages and heights were obtained from the official team roster.

### 2.3 Dependent variables

The CMJ force-time metrics were selected based on previously published research reports (24, 28–30). The dependent variables of interest during the eccentric phase of the CMJ were: braking phase duration and impulse, eccentric duration, peak velocity, and mean and peak force and power. The dependent variables of interest during the concentric phase of the CMJ were: concentric duration, impulse, and peak and mean force and power. In addition, the following outcome and strategy metrics were derived: contraction time, jump height (i.e., impulse-momentum calculation), reactive strength index (RSI)-modified (i.e., jump height divided by contraction time), and countermovement depth. All data was automatically processed via performance analysis software (VALD Performance, Brisbane, Australia). A measured reduction in the system ground reaction force by 20 N indicated the start of the contraction time and ended when the vertical force fell below the 20 N threshold. The eccentric phase was defined as the phase with a negative center of mass velocity. The braking phase was determined from the start of the minimum force until the end of the eccentric phase and the impulse was determined as the area under the ground reaction force curve, excluding the participants' body mass (17, 20, 30).

The raw sprint data was manually processed using the manufacturer-provided software (Version 5.0, Applied Concepts Inc., Dallas, TX, USA) (31). Then, RStudio software (Version 1.4.1106) was used for further data treatment (17, 25) to derive the following dependent variables of interest that demonstrated excellent levels of inter-day and intra-day reliability: maximal and average acceleration, maximal velocity, and time-to-stop (26, 27).

### 2.4 Statistical analysis

Shapiro-Wilk test and Q-Q plots corroborated that the assumption of normality was not violated. Independent *t*-tests were used to examine statistically significant differences for each dependent variable of interest between 3 × 3 (*n* = 10) and 5 × 5 (*n* = 11) basketball players. Hedge's g was used to calculate the magnitude of between-group differences (*g* = 0.2-small effect, *g* = 0.5-moderate effect, *g* = 0.8-large effect) (1, 32). Statistical significance was set *a priori* to *p* < 0.05. All statistical analyses were completed with SPSS (Version 26.0; IBM Corp., Armonk, NY, USA).

## 3 Results

Descriptive statistics, means and standard deviations (x¯ ± SD), for all dependent variables examined in this investigation are presented in Table 1. No statistically significant differences were observed between 3 × 3 and 5 × 5 basketball players in body mass, height, and age (*p* > 0.05). While attaining considerably greater average and maximal decelerations during sprints, the time-to-stop was notably faster for 5 × 5 when compared to 3 × 3 basketball players. However, no significant differences between the two groups in sprint performance were observed in maximal velocity as well as average and maximal acceleration capacities. Both groups revealed similar CMJ performance, with no statistically significant differences being detected in any force-time metrics of interest examined in the present study during both eccentric and concentric phases of the CMJ movement.

**Table 1:** Anthropometric characteristics, sprint performance metrics, countermovement vertical jump force-time variables, and comparison statistics for 3 × 3 and 5 × 5 basketball players. Bolded values represent between-group statistically significant differences (*p *< 0.05).

Variable	3 × 3	5 × 5	*p*-value	ES
Anthropometric characteristics				
Body mass [kg]	100.8 ± 6.6	98.8 ± 9.0	0.311	0.251 (S)
Height [cm]	197.8 ± 3.5	199.4 ± 8.2	0.577	0.249 (S)
Age [years]	23.0 ± 1.5	22.4 ± 1.8	0.434	0.360 (S)
Sprint performance				
Maximal velocity [m/s]	6.37 ± 0.41	6.34 ± 0.29	0.844	0.085 (S)
Average acceleration [m/s^2^]	3.54 ± 0.36	3.35 ± 0.21	0.136	0.653 (M)
Maximal acceleration [m/s^2^]	7.30 ± 0.93	7.69 ± 0.82	0.317	0.446 (M)
Average deceleration [m/s^2^]	−3.20 ± 0.42	−4.01 ± 0.44	**<0**.**001**	1.881 (L)
Maximal deceleration [m/s^2^]	−5.61 ± 0.80	−6.90 ± 0.93	**0**.**003**	1.481 (L)
Time-to-stop [s]	1.68 ± 0.18	1.46 ± 0.18	**0**.**012**	1.222 (L)
CMJ performance				
Braking phase duration [s]	0.313 ± 0.036	0.306 ± 0.038	0.626	0.189 (S)
Braking impulse [Ns]	69.6 ± 10.1	65.8 ± 14.1	0.484	0.307 (S)
ECC duration [s]	0.550 ± 0.085	0.525 ± 0.068	0.461	0.327 (S)
ECC peak velocity [m/s]	−1.24 ± 0.21	−1.30 ± 0.22	0.501	0.278 (S)
ECC mean force [N]	990.3 ± 65.6	954.7 ± 88.8	0.313	0.452 (M)
ECC peak force [N]	2,315.2 ± 193.7	2,282.6 ± 275.9	0.760	0.136 (S)
ECC mean power [W]	609.9 ± 85.0	618.2 ± 169.7	0.891	0.061 (S)
ECC peak power [W]	1,695.2 ± 342.4	1,803.6 ± 488.9	0.567	0.255 (S)
CON impulse [Ns]	282.9 ± 18.7	269.6 ± 30.2	0.244	0.523 (S)
CON duration [s]	0.274 ± 0.021	0.263 ± 0.028	0.329	0.441 (S)
CON peak velocity [m/s]	2.91 ± 0.19	2.88 ± 0.13	0.614	0.186 (S)
CON mean force [N]	2,026.1 ± 144.1	1,977.4 ± 125.3	0.417	0.362 (S)
CON peak force [N]	2,437.2 ± 172.6	2,419.2 ± 191.2	0.823	0.099 (S)
CON mean power [W]	3,098.1 ± 315.3	2,997.0 ± 226.6	0.406	0.371 (S)
CON peak power [W]	5,627.6 ± 564.4	5,343.8 ± 646.0	0.299	0.466 (M)
Contraction time [s]	0.824 ± 0.102	0.782 ± 0.077	0.295	0.468 (M)
Jump height [cm]	40.5 ± 6.0	39.2 ± 3.7	0.562	0.264 (S)
RSI-modified [ratio]	0.511 ± 0.092	0.516 ± 0.063	0.898	0.064 (S)
CMJ depth [cm]	−33.5 ± 4.1	−32.1 ± 4.5	0.459	0.324 (S)

ES, Hedges’ g effect size; CMJ, countermovement vertical jump; RSI, reactive strength index; ECC, eccentric; CON, concentric; S, small effect size; M, moderate effect size; L, large effect size.

## 4 Discussion

To the best of our knowledge, this is the first study to examine force-time metrics during both eccentric and concentric phases of the CMJ and sprint acceleration and deceleration capabilities of top-tier 3 × 3 and 5 × 5 professional basketball players (e.g., national team, NBA, EuroLeague). The results of this investigation reveal no statistically significant differences in any anthropometric or CMJ performance characteristics of interest between the two groups of athletes (*g* = 0.061–0.468). However, prominent differences were found in multiple sport-specific measures of sprint performance, with large effect size magnitudes (*g* = 1.221–1.881). Specifically, while attaining greater average and maximal deceleration, the time-to-stop was considerably lower for 5 × 5 when compared to 3 × 3 basketball players. Overall, besides providing reference values for certain physical performance characteristics, these findings may be used by sports practitioners to better understand sport-specific demands and improve assessment methods and training regimens targeted toward optimizing on-court basketball players' performance.

When comparing body mass and height measurements between 3 × 3 and 5 × 5 basketball players, no statistically significant differences were observed (*p* = 0.311–0.577). Moreover, the between-group difference was small in magnitude (*g* = 0.249–0.251). These findings are in line with previously published research reports examining similar cohorts of basketball athletes (1, 14–18). Although not necessarily novel, they do highlight the importance of the apparent requisite of anthropometric characteristics for success (e.g., height, body mass) in the game of basketball, regardless of the style of play (e.g., 3 × 3 vs. 5 × 5). In a similar manner, a recently published study by Cui et al. (33) examined 3,610 athletes who participated in the 2000–2018 NBA draft and found that height was one of the key variables that differentiated drafted and non-drafted players across all five playing positions (e.g., forwards, guards, centers). So, this information may be beneficial for sports practitioners when trying to identify potential talent and develop adequate physiological profiles of 3 × 3 and 5 × 5 professional male basketball players.

Assessing sprint performance capabilities within basketball populations is one of the standard testing modalities used in basketball-specific settings to assess speed-related metrics, including the players' ability to accelerate, decelerate, and generate maximal sprint velocity (17, 22, 23). However, limited research regarding sprint performance is currently available for 3 × 3 basketball players, especially those participating at professional levels of competition. The findings of the present study provide evidence pertaining to the presence of differences between 3 × 3 and 5 × 5 basketball players in average deceleration velocity (*p* < 0.001), maximal deceleration velocity (*p* = 0.003), and time to stop (*p* = 0.012). Specifically, 5 × 5 athletes demonstrated superior deceleration performance and notably lower time-to-stop values. While further research is warranted on this topic, it is speculated that this observation can be primarily attributed to differences in the number of players on the court as well as court dimensions. When compared to their 3 × 3 counterparts, 5 × 5 basketball players need to avoid more defenders in less space (e.g., 5 vs. 3 defenders) in order to achieve tactical advantages. Thus, deceleration and change-of-direction capabilities might be of greater importance for 5 × 5 basketball players due to sport-specific competitive demands. This information can be beneficial to sports practitioners when collaboratively working to optimize athletes' performance. For the sports coaches, this information may be useful with the selection and design of drills during practice as well as their intensity to mimic the unique demands of each style of basketball competition. For strength and conditioning professionals, understanding the differences in agonistic-antagonistic muscle groups between the two styles of play may contribute to the improvement of training regimens that adequately resemble the athletes' needs. Lastly, for the sports scientist, a further examination of sprint performance may be beneficial for providing deeper insight into the sport-specific demands, including potential risks for injury.

Although a broad spectrum of 5 × 5 basketball players' physical performance characteristics has been examined in the scientific literature over the previous couple of decades (14–18, 29), limited data exists pertaining to 3 × 3 basketball athletes, especially the ones participating in the top-tier professional leagues. Previous research reports have utilized the CMJ to successfully assess neuromuscular performance, jump strategy, and position-specific differences across a broad spectrum of athletes (17, 18, 20, 21). However, to the best of the authors' knowledge, the current study is the first to compare force-time metrics during both eccentric and concentric phases of CMJ between elite 3 × 3 and 5 × 5 male basketball players. The results revealed no statistically significant between-group differences in the neuromuscular (i.e., CMJ) performance parameters of interest (*g* = 0.064–0.525), suggesting that lower-body strength and power are equally important for both competitive styles of play. This is likely due to the need for both 3 × 3 and 5 × 5 athletes to effectively perform similar basketball-specific movements that are founded on CMJ motion (e.g., dunking, shooting, rebounding), which have shown to be of critical importance for securing the desired game outcome (34). However, future research is still warranted to examine fatigue-induced neuromuscular performance changes during practice and official competition as well as how they relate to external loads (i.e., objective quantification of the work performed by the athlete).

As with many investigations conducted on a cohort of professional athletes, one of the limitations of this study is the sample size. In the future, a collaborative effort between coaches, strength and conditioning practitioners, and sports scientists from several basketball organizations can be useful for increasing future sample sizes and addressing this issue. In addition, despite not being a primary objective of this investigation, future research may find it beneficial to examine position-specific differences in both neuromuscular and sporting performance parameters on a similar competitive level (e.g., NBA, EuroLeague). By doing so, further insights may be gained regarding unique differences between position groups in a popular, but under-examined sporting population.

## 5 Conclusion

In conclusion, the findings of the present study suggest that 3 × 3 and 5 × 5 top-tier professional male basketball players tend to display similar neuromuscular performance, as no significant differences were observed in any force-time metric during both eccentric and concentric phases of CMJ movement. However, likely due to competitive demands influenced by the number of players on the court, 5 × 5 showed greater average and maximal deceleration and considerably lower time-to-stop when compared to their 3 × 3 counterparts. In addition, no significant differences were observed in body mass, height, and age between the two groups. Overall, these findings provide reference values that coaches, strength and conditioning practitioners, and sports scientists can use when assessing athletes' CMJ and sprint performance capabilities as well as when developing individually tailored training regimens to mimic on-court competitive demands.

## Data Availability

The raw data supporting the conclusions of this article will be made available by the authors, without undue reservation.
